# Semantic Verbal Fluency Pattern, Dementia Rating Scores and Adaptive Behavior Correlate With Plasma Aβ_42_ Concentrations in Down Syndrome Young Adults

**DOI:** 10.3389/fnbeh.2015.00301

**Published:** 2015-11-18

**Authors:** Laura Del Hoyo, Laura Xicota, Gonzalo Sánchez-Benavides, Aida Cuenca-Royo, Susana de Sola, Klaus Langohr, Ana B. Fagundo, Magí Farré, Mara Dierssen, Rafael de la Torre

**Affiliations:** ^1^Neurosciences Research Program, Integrative Pharmacology and Systems Neuroscience Research Group, IMIM-Institut de Hospital del Mar d’Investigacions MèdiquesBarcelona, Spain; ^2^Departamento de farmacología, Universitat Autònoma de BarcelonaBarcelona, Spain; ^3^Systems Biology Program, Cellular and Systems Neurobiology, Centre for Genomic Regulation (CRG), The Barcelona Institute of Science and TechnologyBarcelona, Spain; ^4^Department of Experimental and Health Sciences, Universitat Pompeu FabraBarcelona, Spain; ^5^Department of Statistics and Operations Research, Universitat Politècnica de Barcelona/BarcelonaTechBarcelona, Spain; ^6^Department of Psychiatry, University Hospital of Bellvitge-IDIBELLBarcelona, Spain; ^7^CIBER Fisiopatología Obesidad y Nutrición (CIBERObn), Instituto Salud Carlos IIIMadrid, Spain; ^8^CIBER de Enfermedades Raras (CIBERER), Instituto Salud Carlos IIIMadrid, Spain

**Keywords:** Down syndrome, Alzheimer’s disease, semantic verbal fluency, switching, Aβ, amyloid precursor protein, communication skills, DMR

## Abstract

Down syndrome (DS) is an intellectual disability (ID) disorder in which language and specifically, verbal fluency are strongly impaired domains; nearly all adults show neuropathology of Alzheimer’s disease (AD), including amyloid deposition by their fifth decade of life. In the general population, verbal fluency deficits are considered a strong AD predictor being the semantic verbal fluency task (SVFT) a useful tool for enhancing early diagnostic. However, there is a lack of information about the association between the semantic verbal fluency pattern (SVFP) and the biological amyloidosis markers in DS. In the current study, we used the SVFT in young adults with DS to characterize their SVFP, assessing total generated words, clustering, and switching. We then explored its association with early indicators of dementia, adaptive behavior and amyloidosis biomarkers, using the Dementia Questionnaire for Persons with Intellectual Disability (DMR), the Adaptive Behavior Assessment System-Second Edition (ABAS-II), and plasma levels of Aβ peptides (Aβ_40_ and Aβ_42_), as a potent biomarker of AD. In DS, worse performance in SVFT and poorer communication skills were associated with higher plasma Aβ_42_ concentrations, a higher DMR score and impaired communication skills (ABAS–II). The total word production and switching ability in SVFT were good indicators of plasma Aβ_42_ concentration. In conclusion, we propose the SVFT as a good screening test for early detection of dementia and amyloidosis in young adults with DS.

## Introduction

Adults with Down syndrome (DS) have a high risk for the development of early onset dementia and invariably develop senile plaques, composed of β-amyloid peptide (Aβ), indistinguishable from the histopathology of sporadic Alzheimer’s disease (AD; Rumble et al., 1989). Plaques can be found in almost all adults from 35–40 years of age (Zigman et al., 2008), and the presence of Aβ oligomers can be detected as early as during fetal development (Teller et al., 1996; Lott and Dierssen, 2010), although the clinical symptoms clearly differ from those observed in AD in general population.

The increase in lifespan in the DS population has made the early detection of dementia of Alzheimer’s type a major objective of researchers and clinicians. In the general population impairments in semantic fluency exist prior to the clinical diagnosis of AD (Vogel et al., 2005). Specifically, patients with AD exhibit important deficits in both semantic and phonemic fluency, being the former the most impaired (Cerhan et al., 2002; Canning et al., 2004; Henry et al., 2004; Taylor et al., 2005). Thus, alterations in clustering and switching abilities during the performance of a semantic verbal fluency task (SVFT) are considered early predictors of the development of AD in the general population (Palmer et al., 2003; Fagundo et al., 2008).

Conversely, even though research on DS has substantiated language and verbal fluency as one of the most impaired domains (Palmer et al., 2003) influencing cognitive-related outcomes and daily living functionality (Edgin et al., 2010; de Sola et al., 2015), there is a paucity of information about the verbal fluency pattern in DS young adults. To our knowledge, only two studies have reported the semantic verbal fluency pattern (SVFP) in DS, one in pediatric population, in which a reduced productivity of words and switching was shown in DS subjects compared to age-matched controls (Nash and Snowling, 2008) suggesting less efficient retrieval strategies. The second one in adult population with learning disabilities (Rowe et al., 2006), showed reduced word production, but the responses were only analyzed accounting for total number of correct words, regardless of performance in retrieval strategies such as clustering and switching.

To date AD conversion in aged DS subjects is mainly analyzed by measuring plasma Aβ concentrations. Several studies have shown increased concentrations of both Aβ_40_ and Aβ_42_ in young DS compared to control population (Mehta et al., 2003; Head et al., 2011) and most found higher concentrations of Aβ_42_ in those DS individuals that were either demented or developed dementia at follow-up (Schupf et al., 2007; Prasher et al., 2010; Coppus et al., 2012). Some correlations have also been found between high Aβ_40_ plasma levels and dementia status, and between increases in Aβ_40_ and decreases of Aβ_42_ and risk of dementia (Schupf et al., 2007, 2010; Head et al., 2011; Coppus et al., 2012). Interestingly, most studies report no correlation between age and Aβ_42_ levels (Prasher et al., 2010; Head et al., 2011).

In the current study, we aimed at characterizing the SVFP including clustering and switching abilities in adults with DS in comparison to age-matched general population. To this aim, we used the SVFT that requires verbal abilities, search and retrieval skills, adequate processing speed, and the capacity to inhibit inappropriate responses (Henry and Phillips, 2006). The total number of words and the clustering, which measures the way these words are grouped by different semantic categories (i.e., pets, farm, aquatic animal etc.), provide an indirect measure of the organization of semantic representations. On the other hand, the use of retrieval strategies, such as switching from one semantic category group of words to a new one, yields information about the set shifting ability, an executive skill related to the integrity of the frontal lobes. We then explored the association of SVFT performance with early indicators of dementia, adaptive behavior and amyloidosis biomarkers (Aβ40 and Aβ42).

## Materials and Methods

### Participants

The sample was drawn from the baseline visit of a clinical trial (TESDAD Study ClinicalTrials.gov Identifier: NCT01699711). Participants enrolled in our cross-sectional study (*n* = 50) were young adults (aged 17–34 years) of both genders with DS (complete trisomy 21, mosaic or translocation). Subjects with neurological disease other than DS (epilepsy, cerebral palsy, hemiplegia, central nervous system infection with neurological deficit), relevant medical disease, unstable co-morbid mental disorder (anxiety disorder, depression, obsessive compulsive disorder), or undergoing any treatment that could interfere with cognitive function or alter key biomarker analyzed were excluded from the study. Also, exclusion criteria included subjects with severe language deficit (significant speech and/or comprehension limitations), behavioral disturbances and/or poor level of collaboration during the assessment but no subjects were excluded from the analysis by this criterion.

To determine the gap in cognitive performance between DS subjects and healthy adults a comparison group, matched for age (mean age: 22.6 ± 3.8) was included 59 young healthy adults of both genders. These participants were assessed in previous neuropsychological studies (de Sola et al., 2008; Fagundo et al., 2010). Healthy volunteers were excluded if they had neurological or relevant medical diseases, or if they had been diagnosed with a psychiatric disorder following DSM-IV criteria. Whilst prevailing methodology compares DS subjects to healthy controls of the same “mental age” to provide an index of global level of mental functioning (Edgin et al., 2010; Finestack and Abbeduto, 2010), this perspective is not useful for characterizing specific capacities (Costanzo et al., 2013; de Sola et al., 2015).

The study was conducted in accordance with the Declaration of Helsinki and Spanish laws concerning data privacy. The protocol was approved by the Ethical Committee of the Parc de Salut Mar of Barcelona (CEIC-PSMAR). Upon arrival at the research center (Hospital del Mar Medical Research Institute-IMIM), participants, parents and legal guardians (in case of legal incapacitation) were informed of the ensuing protocol and they gave their written informed consent before participating.

### Procedure

#### Semantic Verbal Fluency Pattern

We used the SVFT (Benton et al., 1976) as a measure of semantic memory and executive functioning. Three outcome variables were obtained: (i) the total number of correctly generated words in 60 s, and the percentage of words generated every 15 s; (ii) errors committed including intrusions (words not belonging to the specified semantic category), perseverations and repetitions (same words or same words with different endings); and (iii) clustering and switching measures that were obtained to determine the strategies used to perform the task. Mean cluster size was the main dependent variable for clustering, whereas number of switches was the main dependent variable for switching (Troyer et al., 1997; Troyer, 2000). A cluster was defined as any series of two or more successively produced words belonging to the same semantic subcategory, determined *a priori* (Fagundo et al., 2010). Cluster size was computed by adding up series of words from the same subcategory starting from the second word within each cluster (i.e., a three-word cluster has a size of two). The number of switches was defined and computed as the number of times the participant changed from one cluster to another. Two clusters may also be overlapping, for example, from “farm animals” to “birds” in “cow–pig–chicken–pigeon–eagle.” Here, one switch is made between the cluster “cow–pig–chicken” and “chicken–pigeon–eagle.” The computation of number of switches included single-word clusters. An inter-rater reliability analysis was performed and the reliability studied by means of the intra-class correlation coefficient (ICC) was high with values ranging from 0.89–0.98.

#### Intellectual Quotient IQ

The intellectual quotient estimation was assessed with The Kaufman Brief Intelligence Test (Kaufman and Kaufman, 1990).

#### Functional Measures

The Adaptive Behavior Assessment System-Second Edition (ABAS-II, adult version; Harrison and Oakland, 2003) for evaluating adaptive skills in people with intellectual disabilities and the Dementia Questionnaire for Persons with Intellectual Disability (Evenhuis et al., 2006) previously named Dementia Questionnaire for persons with Mental retardation. The DMR is a self-reported questionnaire about daily living abilities, which measures specific memory and orientation cognitive skills and social deterioration as a result of dementia and/or severe sensory or psychiatric problems. It consists of 50 items and eight subscales. Combined scores on the first three subscales (Short-term memory, Long-term memory and Orientation) are presented as the Sum of Cognitive Scores (SCS). Combined scores on subscales four through to eight (Speech, Practical skills, Mood, Activity and Interest, and Behavioral disturbance) are presented as the Sum of Social Scores (SOS). Higher scores in DMR reflect a worse state, while higher punctuations in ABAS-II reflect a better adaptive behavior.

Both questionnaires were given to the caregivers for completion while participants completed the neuropsychological testing. We ensured they understood how to complete the questionnaires and solved all doubts before and after completion, and checked that all questions were filled.

#### Plasma Aβ Measurement

Overnight fasting blood samples were collected on site by a qualified nurse, during the morning hours. The blood was drawn into 8 mL Heparin Lithium tubes (B&D, UK), centrifuged at 4°C for 15 min at 3000 rpm, and the plasma was distributed in aliquots and stored at −70°C until analysis. Samples (only for DS subjects) were analyzed for plasma Aβ concentrations, using Inno-bia Plasma Aβ forms (Aβ40 and Aβ42, truncated Aβ40 and Aβ42 not reported) assay (Innogenetics, Fujirebio) following the manufacturer instructions. The plaques were read in a Bio-Plex 200 Systems (Bio-Rad) instrument, and the standard curves were fitted using the provided software (Bioplex Manager 6.1).

#### Statistical Analysis

Results are described by means of measures of both central tendency (mean and median) and variability (standard deviation and range) for numeric variables, and absolute and relative frequencies for categorical variables. In the case of the IQ, only the median is reported because no distinction is made of values below 40. The differences between DS and healthy groups with respect to semantic verbal fluency performance are quantified by means of the standardized mean difference (Cohen’s *d*). The computation of all correlations of interest was done using Pearson’s correlation coefficient. ANCOVA models were used to study the associations in DS between semantic verbal fluency outcomes, other cognitive and functional measures, and AD biomarkers, on one hand, and gender, IQ, and age, on the other hand. For these analyses, the IQ was categorized into two groups: mild/moderate (*IQ* ≥ 40) and severe (*IQ* < 40) within the range of ID level.

Statistical significance was set at 0.05. All statistical analyses were performed using the statistical software packages SPSS (Version 18.0; SPSS Inc., Chicago, IL, USA) and R (Version 3.2.1; The R Foundation for Statistical Computing, Vienna, Austria).

## Results

### Descriptive Demographic and Clinical Data of the Participants

In our DS sample, 48% individuals were male and the mean age was 23.6 years (standard deviation (SD): 4.5 years; range: 17–34 years). The median IQ was 41 (38% with IQ less than 40) and a maximum IQ of 70, whereas the mean K-BIT standardized score was 103 (SD: 14.9; range: 80–151). In terms of gender, the median IQ among males was 40 (IQ less than 40: 37.5%; maximum: 66) and among females 41.5 (IQ less than 40: 38.5%; maximum: 70), whereas the mean K-BIT standardized scores were 101 (SD: 15.6; range: 80–144) and 105 (SD: 14.3; range: 80–151), respectively. Concerning the DS karyotypes, the sample showed the usual proportion for this population, with most individuals with full trisomy 21 (48 simple trisomies, one translocation, and one mosaic). Regarding Aβ plasma concentrations, the mean Aβ_40_ concentration was 270.9 pg/mL [SD: 50.8; range: 174–439.3] and the mean Aβ_42_ was 41 pg/mL [SD: 10; range: 21.5–60.9].

### Semantic Verbal Fluency Performance in DS Individuals Compared to Standard Norms

Descriptive analyses, Cohen effect size differences (*d*), and confidence intervals (95% CI) of fluency task performance in DS individuals and age-matched standard norms are summarized in Table 1. Our results show that in DS switching correlated more strongly than clustering with the total number of words generated (See Table 2). We found no correlation between the percentage of words produced in the first 15 and last 45 s, with the total number of words. The mean percentage of words produced in the first 15 s was 37.8%.

**Table 1:** **Cognitive performance in DS individuals compared to standard norms**.

	Down syndrome		Reference standard norms		Standardized mean differences	
Verbal fluency	Mean *(SD)*	Range *(min–max)*	Mean *(SD)*	Range *(min–max)*	*d*	*95%-CI*
Number of correct words in 60’	9.4 (4.1)	1–20	25.1 (5.7)	11–38	−3.13	[−3.69, −2.57]
Percentage of correct words 0–15’	39.2 (16.6)	0–100	39.6 (7.9)	25–54	−0.03	[−0.46, 0.4]
Percentage of correct words 16–30	28.1 (12.3)	0–50	22.7 (6.5)	14–39	0.53	[0.09, 0.96]
Percentage of correct words 31–45	16.4 (12.4)	0–50	18.6 (5.8)	5–32	−0.22	[−0.65, 0.21]
Percentage of correct words 46–60	17.1 (12.0)	0–60	18.6 (9.5)	0–46	−0.14	[−0.56, 0.29]
Number of switches	4.3 (2.5)	0–13	7.4 (2.1)	3–11	−1.4	[−1.82, −0.97]
Mean cluster size	1.1 (0.8)	0–3.3	2.8 (0.9)	1.4–6.6	−1.93	[−2.39, −1.46]

*The standardized mean differences are calculated using Cohen’s d. Age range: DS: 17–34, Reference standard norms 18–33 Sample size: DS: n = 51, Reference Standard norm: n = 59*.

**Table 2:** **Correlation between fluency strategies and the total number of words produced (Pearson’s correlation coefficient)**.

	Total correct words			
	Down syndrome		Reference standard norms	
	Correlation [95%-*CI*]	*p*-value	Correlation [95%-*CI*]	*p*-value
Number of switches	0.73 [0.57, 0.84]	<0.001	0.17 [−0.1, 0.41]	0.244
Mean cluster size	0.3 [0.02, 0.53]	0.039	0.49 [0.26, 0.67]	<0.001
Percentage of animals in the first 15 s	0.03 [−0.25, 0.31]	0.84	−0.54 [−0.73, −0.25]	0.001
Percentage of animals in the last 45 s	0.11 [−0.18, 0.38]	0.453	0.51 [0.22, 0.72]	0.001

On the contrary, in age-matched healthy population there is a stronger correlation between clustering and the total number of generated words, while there is no correlation between switching and the total number of words produced. Besides, we found a negative correlation between the percentage of words produced in the first 15 s (mean *percentage* = 39.6) and the total number of words, and a positive correlation between the percentage of words produced during the last 45 s and the total number of words, indicating a more extensive lexicon in this population.

### Association between IQ, Gender and Age, and Semantic Verbal Fluency Outcomes in DS

ANCOVA models were applied to analyze the association between the IQ, gender, and age and the semantic verbal fluency performance of DS individuals. As shown in Table 3, no statistically significant associations were found between IQ, age and gender, and the verbal fluency pattern in the DS group.

**Table 3:** **Association between the verbal fluency pattern and the intellectual quotient (IQ), sex and age in DS individuals**.

	IQ (<40 vs. ≥40)		Sex (Women vs. men)		Age	
Verbal fluency outcomes	Estimate *(SE)*	*p*	Estimate *(SE)*	*p*	Estimate *(SE)*	*p*
Number of correct words in 60 s	−1.04 (1.18)	0.386	0.45 (1.17)	0.704	0.18 (0.13)	0.190
Number of switches	0.25 (0.73)	0.736	0.44 (0.72)	0.547	0.04 (0.08)	0.663
Mean cluster size	−0.36 (0.23)	0.112	−0.07 (0.22)	0.750	0.02 (0.03)	0.488

*Parameter estimates, standard errors (SE), and p-values are obtained from ANCOVA models*.

### Association between IQ, Gender, and Age and AD Biomarkers in DS

ANCOVA models were applied to analyze the association between IQ, gender, and age, on one hand, and Aβ_40,_ Aβ_42,_ Aβ_42/40_ plasma concentrations of DS individuals, on the other hand. We found a statistically significant association between IQ and Aβ_40_. The negative parameter estimate indicates lower Aβ_40_ concentrations among DS individuals of the same age and sex with an *IQ* < 40 compared with those with an *IQ* ≥ 40 (Table 4).

**Table 4:** **Association between Aβ concentrations and intellectual quotient (IQ), sex, and age in DS individuals**.

	IQ (<40 vs. ≥40)		Sex (Women vs. men)		Age	
Aβ concentrations	Estimate *(SE)*	*p*	Estimate *(SE)*	*p*	Estimate *(SE)*	*p*
Aβ_42_	−2.05 (2.97)	0.495	2.12 (2.94)	0.475	−0.21 (0.33)	0.527
Aβ_40_	−38.2 (14.6)	0.012	19.9 (14.2)	0.167	−0.26 (1.59)	0.873
Aβ_40/42_	0.007 (0.013)	0.564	0.004 (0.012)	0.758	−0.0002 (0.001)	0.893

*Parameter estimates, standard errors (SE), and p-values are obtained from ANCOVA models*.

### Associations of Aβ_42_ Concentration with Cognitive and Dementia Rating Functional Outcomes

**Figure 1:**
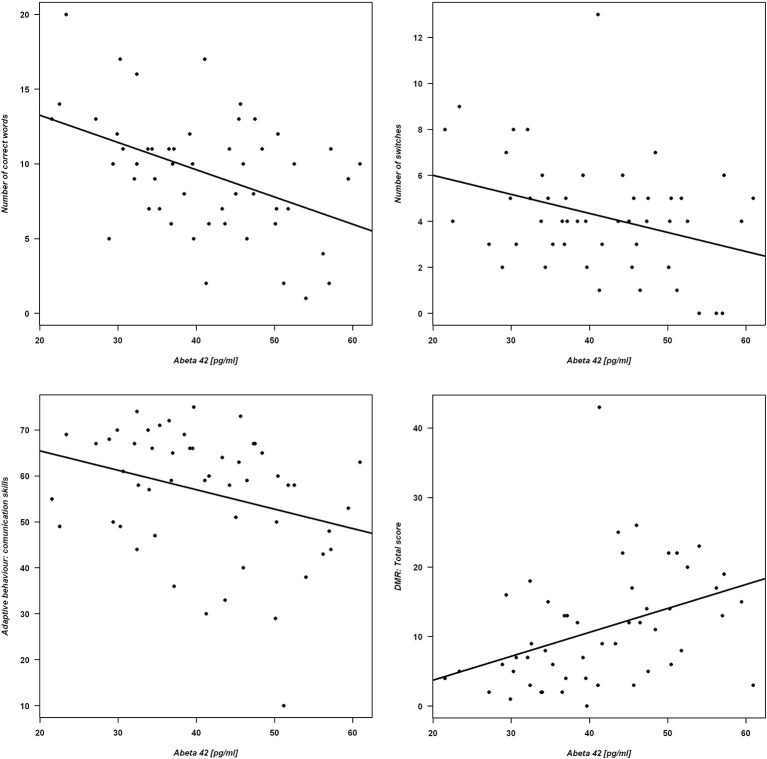
**Verbal fluency and functional measures as a function of Aβ_42_ concentration.** Correlations are shown for Aβ_42_ and Upper panel: number of correct words (left) and number of switches (right). Lower panel: ABAS adaptive behavior (left) and DMR total score (right). The figures include the regression lines from the corresponding linear regression models.

ANCOVA models were applied to analyze the association between Aβ_42_ concentration and both semantic verbal fluency outcomes and functional state among DS individuals. The models were adjusted for IQ, sex, and age (Table 5). Individuals with higher concentrations of Aβ_42_ produced lower number of correct words and lower number of switches. Regarding adaptive behavior, subjects with higher Aβ_42_ plasma concentrations had lower scores in the subscale “Communication skills” of the ABAS questionnaire. Concerning dementia rating, higher Aβ_42_ plasma concentrations were associated with higher DMR total score; see Figure 1 for graphical representations of the statistically significant associations.

**Table 5:** **Association between Aβ_42_ concentration and cognitive and functional measure**.

	Estimate *(SE)*	*p*
**Cognitive performance in SVFT**		
Number of correct words in 60’	−0.187 (0.052)	<0.001
Number of switches	−0.085 (0.035)	0.018
Mean cluster size	−0.006 (0.011)	0.582
**Functional outcomes**		
ABAS adaptive behavior: communication skills	−0.532 (0.158)	0.001
DMR total score	0.366 (0.113)	0.002

*Parameter estimates, standard errors (SE), and p-values are obtained from ANCOVA models adjusted for IQ, age, and sex*.

### Correlation between Fluency Measures and Functional Outcomes

The total number of words produced in 1 min and switching have both a positive correlation with communication skills and a negative correlation with the DMR total score. Furthermore, the total number of words produced in 1 min is positively correlated with the ABAS total score (Table 6).

**Table 6:** **Correlation between cognitive and functional variables measured using Pearson’s correlation coefficient**.

	DMR total		ABAS total		ABAS communication skills	
	Correlation [95%-*CI*]	*p*-value	Correlation [95%-*CI*]	*p*-value	Correlation [95%-*CI*]	*p*-value
Number of correct words in 60’	−0.5 [−0.68, −0.25]	<0.001	0.32 [0.05, 0.55]	0.024	0.45 [0.2, 0.65]	0.001
Number of switches	−0.39 [−0.6, −0.12]	0.006	0.21 [−0.07, 0.46]	0.144	0.28 [0.01, 0.52]	0.046
Mean cluster size	−0.14 [−0.41, 0.14]	0.328	0.13 [−0.15, 0.4]	0.36	0.22 [−0.06, 0.47]	0.127

## Discussion

Our study has found an association between the SVFP, dementia rates, and adaptive behavior related to communication skills in young adults with DS. Moreover, worse semantic fluency, higher dementia rates, and poor adaptive behavior and communication skills were associated to higher plasma concentrations of an AD biomarker (Aβ_42_).

The observed associations between cognitive, functional, and biological parameters suggest that SVF assessment could be used a screening test for early detection of early symptoms of dementia DS. Furthermore, our study shows for the first time clear differences in the SVFP of a DS young adult population compared to healthy age-matched individuals.

### Fluency Deficits in Young Down Syndrome Adults

Impairment of verbal fluency, as estimated by lexical knowledge, is a feature of DS (Rowe et al., 2006). Our results showed a reduction of switching and cluster size as compared to the age-matched group, possibly due to a worse semantic knowledge. This profile is similar to the so called dysexecutive syndrome, described as a common pattern of dysfunction in executive functions such as planning, abstract thinking, flexibility, and behavioral control (Wilson et al., 1998). A dysexecutive syndrome has already been reported in DS (Rowe et al., 2006; Lanfranchi et al., 2010; de Sola et al., 2015), and has been related to the reduced volume of the prefrontal cortex reported in neuroimaging studies (Raz et al., 1995; White et al., 2003; Carducci et al., 2013), in particular affecting the anterior cingulate gyrus, medial, and dorsolateral prefrontal cortices (Contestabile et al., 2010; Lott and Dierssen, 2010). These areas actively contribute to mnemonic processing and executive control in euploid individuals (Braver et al., 2001; Wager and Smith, 2003; Blumenfeld et al., 2011), and, thus, the generalized impairment of high order frontal-dependent processes has a negative influence on SVFP which depends on both mnemonic and executive processes.

Similarly to what is observed in healthy population in our DS group the word production decreases significantly with time, although in the DS group we detected the wide individual variability typically shown in the DS population. The production decrease over time can be explained according to the model of lexical organization (Crowe, 1996), which states that there are two types of storages, namely: (1) a long-term storage (“topicon”) which is readily accessible and contains common words and (2) a more extensive lexicon which is searched after the “topicon” is exhausted. Thus, successful performance on a verbal fluency task seems to be subjected to the effectiveness of both automatic and controlled processing (Crowe, 1998; Hurks et al., 2006). In our DS sample, subjects are not differentiating between using automatic processing and instead, they access to the pool of frequently used words, but when this is exhausted, they fail in using controlled attentional searching retrieval processes that involve executive strategies with high impact on total word production, such as switching. Word production in normative age matched population also decreases over time, paired with a high percentage of words produced in the first 15 s as reported in previous studies (Villodre et al., 2006). However, their topicon and lexicon are richer than DS due to better semantic knowledge (clustering) and retrieval strategies (switching).

### Aβ Plasma Concentrations in Young DS Group

Regarding the plasma concentrations of amyloid peptides, few studies have measured the concentrations of such biomarkers in young adults (Mehta et al., 2003; Head et al., 2011), and those were performed in older populations. Compared to them, we obtained higher mean concentrations of Aβ_42_ (41 ± 10 pg/mL), possibly due to the sensitivity of method we used. However, another study performed in younger DS subjects (mean 7.2 ± 3.8) obtained concentrations (31.6 ± 8.2 pg/mL) that were closer to our mean values (Mehta et al., 2007).

Contrary to previous studies in older DS populations (Prasher et al., 2010; Schupf et al., 2010; Head et al., 2011) that report a correlation of Aβ_42_ with age, in ours this is not present suggesting that factors other than age are affecting the Aβ_42_ production.

In light of our results, the impact of these biomarkers and their evolution pattern should be studied throughout adulthood in DS, and not only in the elderly.

### Association between Fluency Performance and Aβ Concentrations

A large subset of aged individuals with DS develop clinical features of AD and some studies have suggested deficits in executive function (Holland et al., 2000; Ball et al., 2006). In AD patients, AD was better predicted by the clustering ability in some reports (Fagundo et al., 2008), although others (Raoux et al., 2008) found a significant decline in switching along the early phase until the clinical diagnosis of AD dementia. In our DS population, switching and the total number of words are the verbal fluency markers that better correlate with the plasma Aβ_42_ concentrations. This observation supports the hypothesis that impaired switching abilities could explain the early decline in semantic fluency performance in an early state of AD. Moreover, the association between AD biomarkers and verbal fluency pattern is supported by the correlation that we found between Aβ concentrations, dementia ratings (DMR), and communication skills. We observed that higher concentrations of Aβ_42_ were associated to lower adaptive behavior and communication skills and higher DMR scores. In accordance, DMR can be considered useful detecting early symptoms of AD in DS. These results would also be in agreement with previous studies linking higher Aβ_42_ plasma concentrations in elderly DS with dementia or the development of dementia (Schupf et al., 2007; Prasher et al., 2010; Coppus et al., 2012). Furthermore, DMR scores were inversely correlated with the SVFP. This is interesting because, in our study, positive correlations were found between communication skills, semantic verbal fluency, and switching, as discussed above. Communication abilities are a compilation of cognitive and social processes such as comprehension, expression, and empathy. Semantic verbal fluency seems to be part of this compilation of abilities involved in verbal expression as forming part of communication skills. In our case, the DS subjects are not demented, but there is a clear correlation between higher Aβ_42_ and worse scores in functional variables that can be used to detect an early dementia state.

### Limitations

The present study has several limitations. First, plasma measurements of Aβ concentrations remain controversial. Their high variability and lack of correlation with the observations of amyloidosis in the brain are some of the reasons leading some researchers to perform their measurements in CSF, that were not performed in our study. In our study, several peripheral tissues and cells, such as muscle and platelets, could be the source of peripheral Aβ (Toledo et al., 2014). However, in the context of clinical trials, as well as in clinical practice in general, it is worth improving the reliability of this blood measurement, as it is much less invasive than CSF extraction, as well as exploring its correlations with early cognitive symptoms of AD. Second, we only compared the SVFT between the DS group and the age-matched group. The rest of assessments as dementia rates, adaptive behavior and Aβ concentrations are lacking a comparative group. Finally, the high number of statistical tests carried out may increase the probability of Type-1 errors. Nonetheless, no correction to control a family-wise significance level of 0.05 has been applied in order not to increase the probability of Type-2 errors.

## Conclusion

Several studies have sought to understand the implications of changes in plasma Aβ concentrations with regard to the development of AD in DS using Mini Mental State Evaluation (MMSE), yet none has looked at the correlations between changes in concentrations and changes SVFP. Our results show an association between SVFP and early AD symptoms and plasma Aβ concentrations supporting the use of SVFT as a useful tool to detect DS subjects who are vulnerable to develop early onset AD.

Our results may be taken as a first step for further studies to find easy and fast non-invasive tools to predict the early onset of AD in DS population.

## Funding

This work was supported by grants, donations and agreements from Fondation Jérôme Lejeune (Paris, France), Instituto de Salud Carlos III FEDER, (PI11/00744), MINECO (SAF2010-19434 and SAF2013-49129-C2-1-R), EU (Era Net Neuron PCIN-2013-060), DIUE de la Generalitat de Catalunya (SGR 2009/1450 and SGR 2009/718).

## Conflict of Interest Statement

The authors declare that the research was conducted in the absence of any commercial or financial relationships that could be construed as a potential conflict of interest.
